# The Uckfield Cottage Hospital

**Published:** 1914-05-23

**Authors:** R. J. Streatfeild

**Affiliations:** Hon. Sec. The Rocks, Uckfield, Sussex


### THE EDITOR'S LETTER-BOX.

### The Uckfield Cottage Hospital.

*To the Editor of* The Hospital.

Sir,—I am obliged to you for calling my attention to the notice of the above in your issue of the 16th inst.

I hope you will allow me space to protest against a notice of this character being published without my being informed of the intention. To have done this would have been common courtesy. The statements are inaccurate both in fact and implication, and unfair both to the hospital and the medical officers.

The hospital is described as a villa residence. This is untrue in fact and implies that it is unsuited for its purpose. It is suggested that the cases treated are few and trivial, and a most unmerited slur is cast on the medical officers by the way the transfers to Brighton are mentioned and especially in the last sentence.

The following facts speak for themselves :

The hospital was built expressly for its present purpose and has never served any other. Since it was opened 1,097 cases have been treated in it, of these twenty-four only have been transferred to the County Hospital and seven to other institutions.

A cottage hospital cannot and does not profess to provide all that can be done in a large institution. Specialists are not found in a small place like Uckfield, and when special treatment is required the medical officers very properly recommend that cases should be sent where it can be obtained. The functions of a cottage hospital are necessarily different from those of the large and more complete institutions to be found in large centres of population, and any suggestions which would increase its usefulness would be gladly considered by my committee, but the notice under consideration is calculated to give a very false idea of its value, and on that ground is both unjust and unfair.—I remain, Sir, yours truly,

R. J. Streatfeild, Hon. Sec.

The Rocks, Uckfield, Sussex, May 16, 1914.

[We welcome Mr. Streatfeild's enthusiasm, as the principal subscriber to his institution. The report of Sir Henry Burdett was a terse statement of what he found when he inspected the Uckfield Cottage Hospital on October 31 last. Mr. Streatfeild's letter does not affect the accuracy of the statements from the hospital administrator's point of view. Taking in order the points raised by Mr. Streatfeild, the buildings may have been erected expressly for their present use, but in fact they follow the lines of an ordinary dwelling-house and are in no sense those of a hospital. There is not even a casualty room or surgery with the usual equipment. As to the cases, a full list is published on pages 8 and 9 of the Report for 1913, which shows that the illnesses were mostly slight, and that the three more serious cases ("stricture," "intestinal obstruction," and "appendicitis") were transferred to the Brighton Hospital. The hospital has been working for thirty-two years, and 1,097 cases treated represent an average of 34.28, a number less than that given by Sir Henry Burdett. An up-to-date cottage hospital, like that at Cirencester or Savernake, for instance, admits all kinds of cases, and is fully equipped for operative and other necessary work. There are an increasing number of this type of cottage hospital, as Sir Henry Burdett's reports have shown. No reflection was made upon the medical staff, as Mr. Streatfeild seems to think. Uckfield Cottage Hospital was inspected, like all the many other hospitals up and down the country, without notice, to enable the inspector to give an exact account of the condition of the hospital and its atmosphere as they in fact were at the time of his visit. Nothing that Mr. Streatfeild urges affects the conclusion at which the inspector arrived from full knowledge of the present position of hospitals and their future outlook. The object of these reports is to convey accurate and reliable information as to the position of each institution inspected.—Ed. The Hospital.]

### What Keeps a Man off the Panel?

*To the Editor of* The Hospital.

